# Stunting in children aged under 2 years living in the eastern part of Indonesia: analysis of the 2010–2018 Indonesia Basic Health Research

**DOI:** 10.1017/S0007114525105771

**Published:** 2026-01-28

**Authors:** Christiana Rialine Titaley, Iwan Ariawan, Ressita Fannia Iwan, Dwi Hapsari Tjandrarini, Nazarina Nazarina, Yekti Widodo, Michael J. Dibley

**Affiliations:** 1 Faculty of Medicine, Pattimura University, Jl. Ir. M. Putuhena, Ambon 97233, Indonesia; 2 Faculty of Public Health, Universitas Indonesia, Kampus UI Depok, Depok, Jawa Barat, Indonesia; 3 National Research and Innovation Agency, Republic of Indonesia, Cibinong Science Center, Jalan Raya Jakarta-Bogor, Bogor 16915, West Java, Indonesia; 4 Sydney School of Public Health, The University of Sydney, Edward Ford Building, A27 Fisher Rd, University of Sydney, Sydney, NSW 2006, Australia

**Keywords:** Stunted growth, Growth and development, Child malnutrition, Nutritional deficiency

## Abstract

This study examined factors associated with stunting in children aged < 2 years in eastern Indonesia. Data were derived from three national cross-sectional surveys of Indonesia. The outcome variable was stunting (low length-for-age) in children aged < 2 years. Nineteen potential predictors from community- to individual-level characteristics were identified. Multilevel analyses were performed, adjusting for cluster sampling with random effects for cluster and strata. We used data from the 2010, 2013 and 2018 Indonesian Basic Health Research. Information from 6076 children aged < 2 years from Nusa Tenggara Barat, Nusa Tenggara Timur, Sulawesi, Maluku and Papua regions were used. We found that the proportion of stunted children aged < 2 years in eastern Indonesia decreased between 2010 and 2018. Significant predictors of stunting included living in West Nusa Tenggara (adjusted OR (aOR) = 1·09; 95 % CI 1·02, 1·16) and East Nusa Tenggara region (aOR = 1·36; 95 % CI 1·28, 1·45), belonging to a household with three or more children aged under 5 years (aOR = 1·32; 95 % CI 1·11, 1·56), being from a poor household (aOR = 1·17; 95 % CI 1·06, 1·30) and born to less educated mother (aOR = 1·26; 95 % CI 1·02, 1·56). Furthermore, stunting were more likely among males (aOR = 1·29, 95 % CI 1·19, 1·40), those aged 12–23 months (aOR = 2·01; 95 % CI 1·65, 2·45), with low birth weight (aOR = 1·91; 95 % CI 1·40, 2·60) and with gestational age < 37 weeks at birth (aOR = 1·14; 95 % CI 1·05, 1·24). Multiple factors contribute to stunting in eastern Indonesia, highlighting the need for comprehensive and targeted initiatives. Poverty reduction, healthcare system improvement, family planning and continued health promotion strategies are necessary to reduce stunting prevalence.

Stunting is a recognised indicator of inadequate child growth and development. It is defined as a height or length below 2 sd below the WHO child growth standard^(1)^. Stunting is an irreversible consequence of insufficient nutrition and recurrent infections within the first 1000 d of a child’s life^(1)^. The long-term consequences of stunting include compromised cognitive and physical growth, decreased productivity, health challenges and heightened susceptibility to chronic illnesses^(2,3)^.

Given the severe impact of stunting, the World Health Assembly has set a global goal to reduce the number of children aged < 5 years with stunted growth by 40 % in 2025^(1)^. In 2020, approximately 22 % (149·2 million) of the children aged < 5 years experienced stunting^(4)^. This figure remains consistent in 2022, with 22·3 % (148·1 million) of the population in that age group being affected^(5)^.

As the fourth most populous country worldwide, Indonesia has contributed 5 % to the global burden of stunting over the past decade^(4)^. An analysis of the 2018 Basic Health Research of Indonesia reported that the proportion of stunted children aged < 2 years living in eastern Indonesia was higher than in other regions^(6)^. This disparity was evident across Indonesian provinces in 2022, with stunting rates ranging from 8 % in Bali (western region) to 35·8 % in Sulawesi Barat Province (eastern region)^(7)^. This difference underscores the importance of examining local risk factors in eastern Indonesia, which might be likely to differ from those in other regions due to its unique challenges, including limited access to healthcare, higher poverty rates and a higher prevalence of infectious diseases such as diarrhoea or acute respiratory infections that could adversely affect children’s nutritional status^(8,9)^.

The Presidential Regulation of the Republic of Indonesia, No. 18 Year 2020, states that the prevalence of stunting should be reduced to 14 % by 2024^(10)^. However, preliminary results of the 2023 Indonesian Health Survey reported a stunting prevalence of 21·5 % in children aged < 5 years^(11)^. This survey also reported that the prevalence of stunting in children aged < 5 years in eastern Indonesia was higher than the national average. Based on this evidence, the target for 2024 may not be achieved. Consequently, the Ministry of National Development Planning/*Bappenas* has set new targets to reduce stunting to 18·8 % by 2024 and 5·0 % by 2045^(12)^. This evidence underlines the urgent need to implement effective and targeted interventions, including nutrition-specific and sensitive measures, particularly in eastern Indonesia, to meet national and international targets^(1)^.

The period from pregnancy to the child’s first 2 years, known as the golden period of life, is crucial, as during this period, children undergo significant growth and development^(13)^. Children aged under 2 years represent the most critical age group for stunting surveillance, as growth faltering typically begins during this period and is largely irreversible after age of 2 years^(14)^. This period is also a critical window when interventions are most effective in preventing or reversing stunting. Therefore, using data from the 2010, 2013 and 2018 Indonesian Basic Health Research, our study aimed to identify the factors contributing to stunting among children aged < 2 years, living in eastern Indonesia. The findings of this analysis will provide valuable insights for policymakers and programme managers to develop targeted interventions and policies to reduce and prevent stunting in this region, which is particularly affected by this issue. Additionally, this analysis will help fill the gap in understanding the specific risk factors for stunting in eastern Indonesia, where unique challenges may contribute differently to stunting than in other regions of the country. By focusing on this age group, our analysis captures the window of greatest vulnerability and opportunities for effective intervention.

## Methods

### Data source and survey design

The data used in this study were obtained from the 2010, 2013 and 2018 Indonesian Basic Health Research conducted by the National Institute of Health Research and Development of the Ministry of Health, Republic of Indonesia^(15–17)^. We pooled data from these three surveys into a single analytic dataset. Each survey was a nationally representative, repeated cross-sectional survey conducted using a multistage systematic random sampling design. To account for differences across survey years, we included the survey year as a covariate in the analysis. This approach enabled us to assess both overall associations and temporal changes in stunting among children aged under 2 years in eastern Indonesia.

Since 2007, the Ministry of Health has conducted a 5-year cross-sectional survey to collect baseline data and determine health-related indicators to assess public health at the district, provincial and national levels. The survey evaluated community health at various levels and identified significant changes in health. The sample sizes in the 2013 and 2018 surveys allowed for estimations up to the district level, whereas the 2010 survey provided only provincial-level data. Information regarding the sample size and census block for each survey is provided in online Supplementary Table 1.

**Figure 1.**
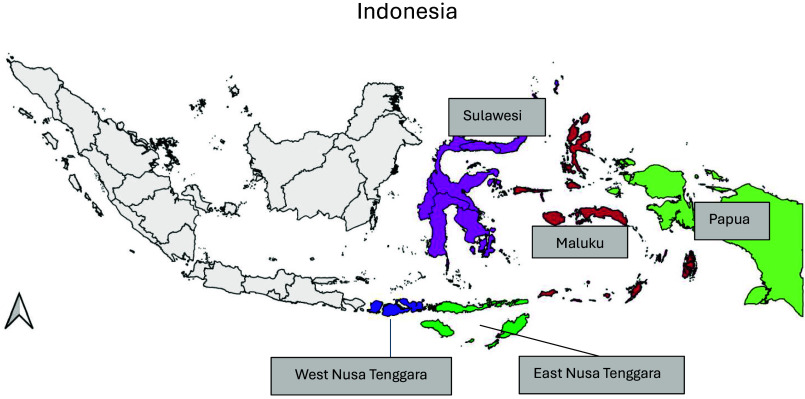
Regions in eastern Indonesia.

The 2010 Indonesian Basic Health Research included a representative sample of households from thirty-three provinces and 441 districts/cities (online Supplementary Table 1). However, the number of districts increased in the 2013 and 2018 surveys, resulting in data being available only for the new districts and not present in the earlier 2010 survey. Detailed survey methodology has been explained elsewhere^(15–17)^. For this analysis, we used data from 6076 respondents (1379 in 2010, 3738 in 2013, and 959 in 2018) who had children aged < 2 years and lived in eastern Indonesia, specifically from the provinces of Nusa Tenggara Barat, Nusa Tenggara Timur, Sulawesi, Maluku and Papua (Figure 1).

### Variables

Based on the theoretical framework developed by the WHO for childhood stunting^(18)^, this analysis included different variables to assess the association between potential predictors available in the survey dataset and stunting in children aged < 2 years.

The main outcome of this analysis was stunting (low length-for-age) in children aged < 2 years. A child was considered stunted if their length-for-age Z-score (HAZ) was below –2 sd ^(19)^. In this survey, a specialised multifunction stadiometer, designed to measure adult height and baby length, was used to determine the child’s body length. The stadiometer had a maximum measuring capacity of 135 cm for young children and a precision of 0·1 cm. Recumbent length was measured in children aged < 2 years. Length-for-age compares a child’s measured length with the average length of healthy children of the same age and sex and is typically quantified as z-scores. Stunted children were assigned a score of 1, and non-stunted children were assigned a score of 0.

This analysis included nineteen potential predictors of stunting, classified into six categories: survey year, community, household and housing, maternal and paternal, antenatal care and child characteristics. Community characteristics included region and type of residence. Household and housing characteristics included the number of children aged < 5 years, total household members, source of drinking water and household wealth index. The household wealth index was constructed using principal component analysis based on twelve variables: (1) main source of drinking water, (2) type of cooking fuel, (3) ownership of toilet facilities, (4) type of toilet, (5) waste disposal method, (6) source of household lighting, (7) motorcycle ownership, (8) television ownership, (9) water heater availability, (10) 12 kg gas cylinder ownership, (11) refrigerator ownership and (12) car ownership.

Maternal and paternal characteristics included evaluations of maternal and paternal education, maternal and paternal employment, and maternal age at childbirth. Antenatal care services included the number of antenatal care visits and the number of iron/folic acid supplements consumed during pregnancy. According to the antenatal care guidelines from the Ministry of Health of the Republic of Indonesia, which adopted the WHO recommendations, antenatal care should be conducted at least six times – twice during the first trimester, once during the second trimester and three times during the third trimester^(20)^. Finally, the child characteristics included sex, breast-feeding status, time of breast-feeding initiation after birth, age at the time of the interview, birth weight and maternal age at the beginning of pregnancy.

All variables were obtained from the structured questionnaires of the Indonesian Basic Health Research Survey. The definitions and categorisations of these variables were based on the original questionnaires and are listed in Table 1.

**Table 1.** The frequency distribution of variables analysed in this study by year of survey and stunting status, Indonesia Basic Health Research 2010–2018

Variables	2010		2013		2018		Total		Stunting	
	*n*	%	*n*	%	*n*	%	*n*	%	%	*P* *
Year										< 0·001
2010	1379	100	–	–	–	–	1379	22·7	43·5	
2013	–	–	3738	100	–	–	3738	61·5	38·4	
2018	–	–	–	–	959	100	959	15·8	29·1	
Community characteristics										
Region										< 0·001
West Nusa Tenggara	188	13·7	474	12·7	150	15·7	813	13·4	34·9	
East Nusa Tenggara	208	15·1	557	14·9	138	14·4	903	14·9	43·9	
Sulawesi	678	49·2	1991	53·3	507	52·9	3176	52·3	34·9	
Maluku	107	7·8	326	8·7	74	7·8	508	8·4	35·4	
Papua	197	14·3	389	10·4	89	9·3	676	11·1	35·9	
Type of residence										0·007
Urban	438	31·8	1225	32·8	332	34·7	1995	32·8	33·4	
Rural	941	68·3	2513	67·2	626	65·3	4080	67·2	37·9	
Household and housing characteristics										
Number of children under 5 years of age living in the household										< 0·001
1	839	60·8	2511	67·2	869	90·7	4219	69·4	34·8	
2	449	32·6	1073	28·7	76	8·0	1598	26·3	39·8	
3+	91	6·6	155	4·1	13	1·3	259	4·3	42·2	
Number of household members										0·225
2–4	519	37·7	1465	39·2	387	40·4	2372	39·1	35·4	
5–7	640	46·4	1867	49·9	448	46·8	2955	48·6	37·0	
8+	219	15·9	406	10·9	123	12·8	748	12·3	37·2	
Type of source of drinking water										0·047
Improved	1035	75·1	3013	80·6	810	84·5	4857	80·0	35·7	
Unimproved	337	24·5	726	19·4	149	15·5	1212	20·0	39·2	
Household wealth index										< 0·001
Rich	311	22·6	1118	29·9	217	22·7	1646	27·1	32·1	
Moderate	242	17·6	639	17·1	218	22·7	1098	18·1	35·6	
Poor	825	59·9	1982	53·0	235	24·6	3043	50·1	39·8	
Maternal and paternal characteristics										
Maternal education										< 0·001
Academy	110	8·0	325	8·7	138	14·5	573	9·4	30·6	
High school	319	23·1	969	25·9	320	33·4	1607	26·5	32·6	
Middle school	280	20·3	755	20·2	197	20·5	1232	20·3	37·3	
No education/up to primary	669	48·6	1613	43·1	303	31·6	2585	42·6	39·6	
Paternal education										< 0·001
Academy	105	7·6	309	8·3	124	13·0	538	8·9	32·2	
High school	300	21·8	1045	28·0	327	34·2	1672	27·5	33·7	
Middle school	229	16·6	620	16·6	167	17·5	1016	16·7	38·9	
No education/up to primary	744	54·0	1482	39·6	340	35·5	2565	42·2	37·8	
Maternal employment										0·002
Housewife	598	43·4	2373	63·5	528	55·1	3499	57·6	36·7	
Working	781	56·6	1288	34·5	431	44·9	2500	41·2	36·0	
Paternal employment										0·001
Not working	61	4·4	160	4·3	27	2·8	247	4·1	34·2	
Working	1318	95·6	3296	88·2	931	97·2	5545	91·3	36·4	
Duration of gestation at childbirth										< 0·001
37–40 weeks	1178	85·5	1865	49·9	826	86·2	3869	63·7	35·5	
< 37 weeks	55	4·0	1624	43·4	63	6·5	1742	28·7	39·3	
> 40 weeks	31	2·2	249	6·7	24	2·5	304	5·0	35·8	
Maternal age at childbirth										0·006
20–29 years	668	48·4	1945	52·0	344	35·9	2957	48·7	36·9	
< 20 years	115	8·3	377	10·1	76	8·0	568	9·4	40·8	
30–39 years	465	33·8	1191	31·9	214	22·4	1871	30·8	35·4	
> 40 years	48	3·5	148	4·0	31	3·3	228	3·8	40·6	
Antenatal care (ANC) services										
Number of ANC visits										< 0·001
6 or more ANC visits	28	2·0	43	1·2	49	5·1	120	2·0	32·6	
1–5 ANC visits	590	42·8	1580	42·3	319	33·3	2489	41·0	38·7	
No ANC visit	228	16·5	312	8·3	35	3·6	574	9·4	37·4	
Number of iron/folic acid tablets consumed during pregnancy										< 0·001
90 or more tablets	148	10·7	1093	29·2	234	24·4	1475	24·3	34·6	
< 90 tablets	700	50·8	1612	43·1	597	62·3	2909	47·9	36·8	
None	370	26·8	494	13·2	127	13·3	991	16·3	37·2	
Could not remember	133	9·7	539	14·4	1	0·1	673	11·1	37·8	
Child characteristics										
Child sex										< 0·001
Female	646	46·9	1854	49·6	474	49·4	2974	49·0	34·2	
Male	733	53·1	1884	50·4	485	50·6	3101	51·0	38·6	
Ever breastfed										< 0·001
Ever breastfed	1241	90·0	3484	93·2	883	92·1	5609	92·3	36·4	
Never breastfed	133	9·6	254	6·8	75	7·9	462	7·6	37·3	
Child age										< 0·001
< 12 months	642	46·6	1745	46·68	458	47·8	2845	46·8	28·1	
12–23 months	737	53·5	1993	53·32	501	52·2	3231	53·2	43·7	
Birth weight										< 0·001
>= 2500 grams	846	61·3	1862	49·8	532	55·5	3239	53·3	33·8	
< 2500 grams	72	5·2	120	3·2	40	4·1	232	3·8	49·2	
Don’t know	461	33·4	120	47·0	387	40·4	2604	42·9	38·5	

All estimates are weighted for sampling probabilities.

* *P* was derived from the *χ* ^2^ test.

### Data analysis

We used descriptive statistical methods in the initial phase to analyse all variables involved. Bivariate analysis was conducted to explore the distribution of these variables across the stunting status. Subsequently, a multilevel analysis was performed, which included two sequential models that incorporated random intercepts. First, a null model (also known as an empty model) was developed to assess the role of districts and provinces without adjusting for all potential predictors at the individual, household and community levels. We computed the median OR for each level to measure its association with stunting status. Subsequently, model 1 was developed to investigate the association of variations at the district and province levels, along with the characteristics at the community, individual and household levels, with stunting status, with adjustments made for each factor. In this model, we used adjusted OR (aOR) to measure the association. We applied a backward elimination process to discard any individual or household characteristics that were not significantly associated with the study outcome, using a significance level of 0·05. Two variables, type of residence (urban/rural) and household wealth index, were chosen *a priori* and retained in the final model, regardless of their significance level.

The final model provided all the aOR and 95 % CI for the predictors. This analysis accounted for the complexity of the sample design. Multilevel models were constructed using Stata/MP software (version 14.2; StataCorp) with the melogit routine.

## Results

**Figure 2.**
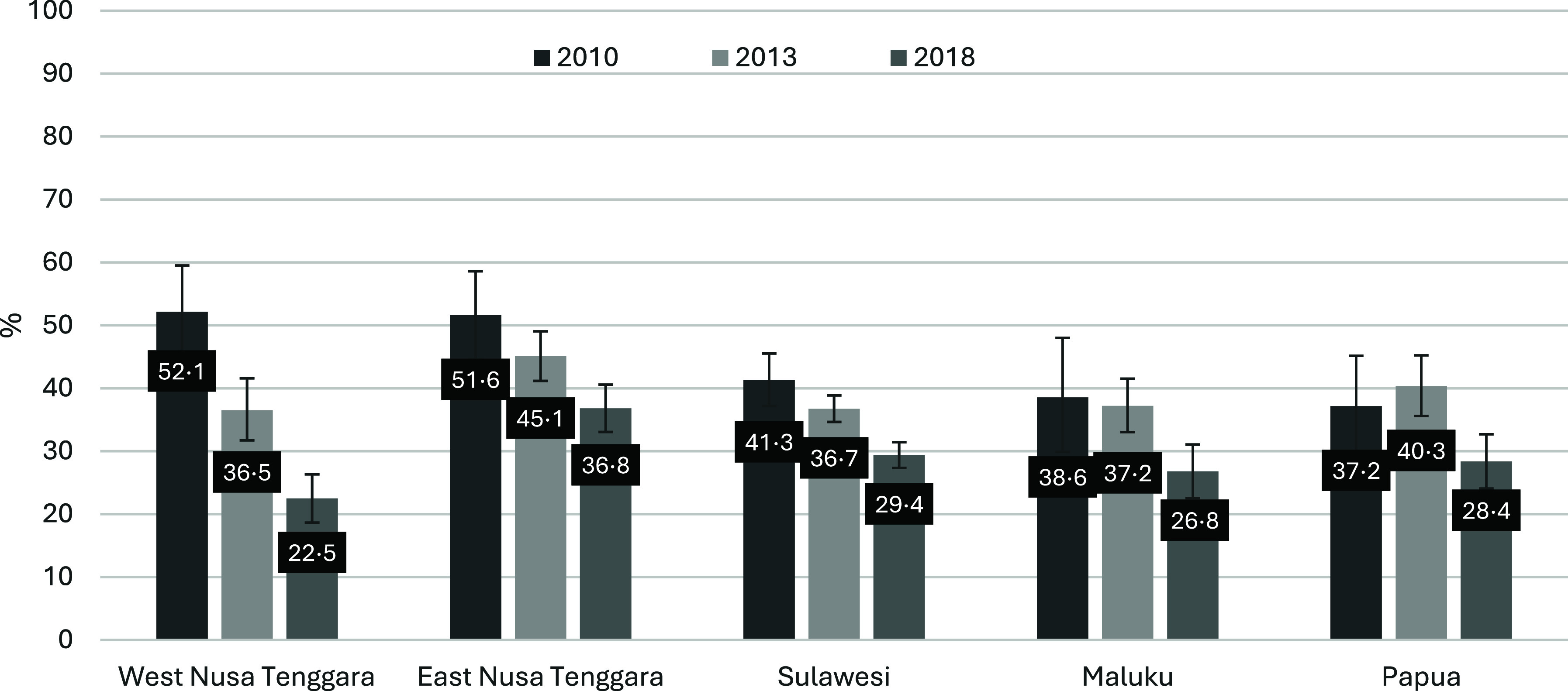
Percentage of stunted children under 2 years of age in Eastern Indonesia by year of survey, the Indonesia Basic Health Research 2010–2018.

Of the 6075 children aged < 2 years analysed in this study, 37·85 % (95 % CI 36·67, 39·04 %) demonstrated stunted growth. Figure 2 shows the proportion of stunted children under 2 years of age from eastern Indonesia in the 2010–2018 surveys. In 2010, the percentage of stunting was 43·51 % (95 % CI 40·62, 46·45) and reduced to 29·09 % (95 % CI 27·61·61, 30·62) in the 2018 survey. The highest reduction was observed in West Nusa Tenggara, from 52·14 % to 22·49 %. The smallest decrease was observed in Papua (from 37·17 % to 28·38 %). The stunting rates by district and year of the survey for each region are shown in online Supplementary Figures 1–5. The prevalence of stunted children under 2 years of age ranged from 16·1 % to 27·7 % in West Nusa Tenggara, 16·9–57·5 % in East Nusa Tenggara, 4·3–65·4 % in Sulawesi, 8·0–45·1 % in Maluku and 0–67·5 % in Papua Region.

Table 1 shows the distribution of all respondents by community level, household and housing, maternal and paternal care services, antenatal care services and children’s characteristics. The prevalence of stunting was highest in the East Nusa Tenggara Region. The prevalence was higher in households with three or more children under 5 years of age (42·2 %), households with a low wealth index (39·8 %), children aged 12–23 months, infants weighing less than 2500 g at birth and infants delivered before 37 weeks of maternal age.

The results of the multilevel model of factors associated with stunting in children aged < 2 years in eastern Indonesia are presented in Table 2. For bivariable multilevel modelling, a significant association was observed between stunting and the year of the survey, region, type of residence, number of children aged < 5 years in the household, source of drinking water in the household, household wealth index, maternal and paternal educational status, maternal age at childbirth, number of antenatal care visits, number of iron/folic acid supplements used during pregnancy, child sex, age at the time of interview, birth weight and maternal age at childbirth.

**Table 2.** Factors associated with stunting in children under 2 years of age in eastern Indonesia, Indonesia Basic Health Research 2010–2018

Variables	Bivariable			Multilevel model			Null model*		
	OR	95 % CI	*P*-value^†^	OR	95 % CI	*P* ^†^	OR	95 % CI	*P* ^†^
Year									
2010	1·00			1·00					
2013	0·81	0·68, 0·97	0·024	0·82	0·67, 1·01	0·050			
2018	0·53	0·42, 0·67	< 0·001	0·56	0·46, 0·68	< 0·001			
Community characteristics									
Region									
Sulawesi	1·00			1·00					
West Nusa Tenggara	1·03	0·95, 1·11	0·448	1·09	1·02, 1·16	0·007			
East Nusa Tenggara	1·45	1·34, 1·57	< 0·001	1·36	1·28, 1·45	< 0·001			
Maluku	0·99	0·89, 1·09	0·791	0·95	0·87, 1·04	0·299			
Papua	1·07	0·96, 1·20	0·189	1·00	0·86, 1·17	0·967			
Type of residence									
Urban	1·00			1·00					
Rural	1·17	1·04, 1·31	0·007	1·04	0·92, 1·18	0·525			
Household and housing characteristics									
Number of children under 5 years of age living in the household									
1	1·00			1·00					
2	1·26	1·14, 1·41	< 0·001	1·22	1·07, 1·40	0·004			
3+	1·43	1·21, 1·68	< 0·001	1·32	1·11, 1·56	0·001			
Number of household members									
2–4	1·00								
5–7	1·06	0·98, 1·16	0·139						
8+	1·10	0·98, 1·23	0·118						
Type of source of drinking water									
Improved	1·00								
Unimproved	1·14	1·04, 1·25	0·004						
Household wealth index									
Rich	1·00			1·00					
Moderate	1·19	1·05, 1·36	0·008	1·10	0·96, 1·26	0·157			
Poor	1·40	1·26, 1·56	< 0·001	1·17	1·06, 1·30	0·003			
Maternal and paternal characteristics									
Maternal education									
Academy	1·00			1·00					
High school	1·10	0·83, 1·45	0·522	1·06	0·80, 1·40	*0·684*			
Middle school	1·35	1·04, 1·76	0·026	1·22	0·96, 1·56	*0·098*			
No education/up to primary	1·50	1·19, 1·88	0·001	1·26	1·02, 1·56	*0·030*			
Paternal education									
Academy	1·00								
High school	1·08	0·89, 1·30	0·475						
Middle school	1·38	1·18, 1·60	< 0·001						
No education/up to primary	1·30	1·12, 1·51	< 0·001						
Maternal employment									
Housewife	1·00								
Working	0·96	0·86, 1·07	0·467						
Paternal employment									
Not working	1·00								
Working	1·04	0·83, 1·30	0·723						
Maternal age at childbirth									
20–29 years	1·00			1·00					
< 20 years	1·19	1·09, 1·30	< 0·001	1·19	1·10, 1·29	< 0·001			
30–39 years	0·94	0·84, 1·06	0·325	0·94	0·83, 1·06	0·319			
> 40 years	1·18	0·93, 1·50	0·168	1·17	0·91, 1·50	0·232			
Antenatal care (ANC) services									
Number of ANC visits									
6 or more ANC visits	1·00								
1–5 ANC visits	1·35	1·01, 1·80	0·041						
No ANC visit	1·33	0·98, 1·80	0·070						
Number of iron/folic acid tablets consumed during pregnancy									
90 or more tablets	1·00								
< 90 tablets	1·17	0·99, 1·38	0·053						
None	1·25	1·04, 1·48	0·015						
Could not remember	1·22	0·92, 1·62	0·174						
Child characteristics									
Child’s sex									
Female	1·00			1·00					
Male	1·25	1·14, 1·36	< 0·001	1·29	1·19, 1·40	< 0·001			
Ever breastfed									
Ever breastfed	1·00								
Never breastfed	1·06	0·88, 1·28	0·538						
Child’s age									
< 12 months	1·00			1·00					
12–23 months	2·01	1·66, 2·43	< 0·001	2·01	1·65, 2·45	< 0·001			
Birth weight									
>= 2500 g	1·00			1·00					
< 2500 g	1·99	1·53, 2·58	< 0·001	1·91	1·40, 2·60	< 0·001			
Don’t know	1·20	1·14, 1·27	< 0·001	1·08	1·00, 1·16	0·037			
District (MOR)				1·00			1·13		
Province (MOR)				1·23			1·23		

All estimates are weighted for sampling probabilities.

* Model that contains only random effects, without any explanatory (independent) variables.

† *P*-value was derived from multilevel modelling.

The results of the multivariable multilevel modelling (Table 2) showed that the odds of stunting were significantly lower in children aged < 2 years surveyed in 2018 than in those surveyed in 2010 (aOR = 0·56, 95 % CI 0·46, 0·68, *P* < 0·001), after adjusting for other covariates. Of the community characteristics included in our analysis, we found significantly higher odds of stunting in infants from West Nusa Tenggara (aOR = 1·09, 95 % CI 1·02, 1·16, *P* = 0·007) and East Nusa Tenggara (aOR = 1·36, 95 % CI 1·28, 1·45, *P* < 0·001) than in those from the Sulawesi Region.

Among household and housing characteristics, the odds of stunting increased significantly in children living in households with two children aged < 5 years (aOR = 1·22; 95 % CI 1·07, 1·40, *P* = 0·004). The odds were even higher for children living in households with three or more children aged < 5 years (aOR = 1·32; 95 % CI 1·11, 1·56, *P* = 0·001). Moreover, the odds of stunting increased in poor households (aOR = 1·17; 95 % CI 1·06, 1·30, *P* = 0·003).

Maternal education emerged as a significant predictor of stunting among maternal and paternal characteristics. Children born to mothers with no education or only primary school education had higher odds of being stunted (26 %) than those born to mothers with a university degree (aOR = 1·26; 95 % CI 1·02, 1·56, *P* = 0·030). The findings also showed that infants born to mothers aged < 20 years at childbirth had significantly increased odds of stunting (aOR = 1·19, 95 % CI 1·10, 1·29, *P* < 0·001).

**Figure 3.**
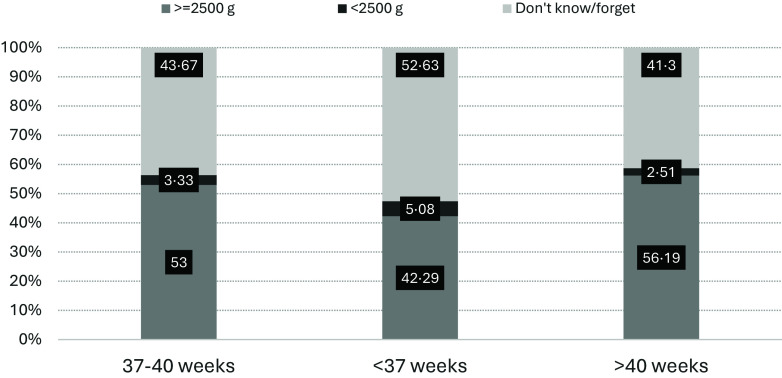
Frequency distribution of children’s birth weight by maternal age at childbirth, the Indonesia Basic Health Research 2010–2018.

Among the child characteristics, male infants had approximately 30 % higher odds of being stunted than female infants (aOR = 1·29, 95 % CI 1·19, 1·40, *P* < 0·001). The odds of stunting in children aged 12–23 months were twice as high as those in infants (< 12 months) (aOR = 2·01; 95 % CI 1·65, 2·45, *P* < 0·001). In children who weighed < 2500 g at birth, the odds of being stunted were 1·91 times higher than those weighing ≥ 2500 g at birth (aOR = 1·91; 95 % CI 1·40, 2·60, *P* < 0·001). However, when the birth weight variable was replaced by the age of pregnancy at childbirth, higher odds of stunting were associated with children born at < 37 weeks of gestation than with those born at 27–40 weeks of gestation (aOR = 1·14; 95 % CI 1·05, 1·24, *P* = 0·001). Our analysis also showed that the odds of stunting were higher in infants whose birth weight was not recorded/not recalled by their mothers (aOR = 1·08; 95 % CI 1·00, 1·16, *P* = 0·037). However, the highest percentage of these infants was found in those born at < 37 weeks’ gestation (52·63 %) (Figure 3).

Table 2 also shows that in the null model (i.e. model that contains only random effects without any explanatory (independent) variables), there was a larger role of the province than the district, as the median OR of province and district were 1·23 and 1·13, respectively. When community-, household- and individual-level factors were added to the null model, the median OR of the province remained unchanged. In contrast, the median OR of the district level was reduced to 1·00.

## Discussion

### Main findings

This analysis demonstrated a high proportion of stunted children aged < 2 years in eastern Indonesia. However, infants whose mothers were interviewed in the 2018 survey were less likely to experience stunting, indicating a reduction in the stunting rate over the survey period. The factors significantly associated with an increased likelihood of stunting were households with a high number of children aged < 5 years, poor households, less-educated mothers, male children, children aged 12–23 months, children weighing less than 2500 g at birth and children born at < 37 weeks of gestation. These findings provide valuable insights for developing targeted interventions and policies to reduce the prevalence of stunting and improve child health outcomes in the eastern region of Indonesia.

### Stunting in children aged < 2 years living in eastern Indonesia

Despite a decrease in the proportion of stunted children in eastern Indonesia over the years, this percentage remains higher than the national target of 14 %^(10)^. This indicates slower progress in the health sector in this region than in central and western Indonesia^(21)^. This slow progress has also been demonstrated in previous studies reporting lower Human Development Index scores in eastern Indonesia than in other regions^(22,23)^. The implementation of nutrition-specific and nutrition-sensitive interventions in eastern Indonesia, as recommended by the government to accelerate stunting reduction efforts, also lags behind other regions. For example, the percentage of women receiving at least six antenatal care services during pregnancy was lower (8·7 %) than that in Western (18·3 %) and central (23·8 %) Indonesia^(24)^. The percentage of pregnant women who took ≥ 90 iron tablets during their last pregnancy was the lowest in Indonesia (28·43 %)^(24)^. This evidence emphasises the need to employ specific strategies to address the distinct challenges faced by communities in eastern Indonesia and a continuous and regular monitoring system to help identify barriers and areas requiring enhancement and ensure that interventions effectively reach those who require them the most.

Our analysis found high stunting rates in some regions, such as West and East Nusa Tenggara, highlighting the urgent need for accelerated interventions targeted at these areas. There was also a wide range of stunting by district within each region and a higher median OR at the district level than at the province level. This suggests that there was more unexplained variation at the district level than at the provincial level, reflecting the contribution of local environmental factors that were not measured in the surveys.

There is a wide range of variations across districts, for example, the accessibility or density of health facilities, which might influence the use of preventive and curative healthcare services. There is also variation in the burden of infectious diseases, such as malaria, dengue, diarrhoeal diseases and intestinal worm infections, which could impair the nutritional status of children. Additionally, the availability of local agricultural produce and market access that affect food security status remain important drivers of undernutrition in many districts. Therefore, stunting reduction interventions should be designed to target district-specific factors; however, further research is required to characterise these factors. Policymakers must consider district-level data to understand unique local challenges and develop customised intervention programmes. This approach can help allocate resources more efficiently and implement strategies tailored to each district’s specific conditions.

### Improving economic status in eastern Indonesia

Our analysis confirmed an association between household economic status and stunting, as reported in other studies^(25,26)^. Household economic constraints often lead to inadequate access to nutritious foods that are essential for proper growth and development and restricted access to healthcare services. Consequently, the increased prevalence of illnesses may hinder nutrient absorption and exacerbate growth deficiencies. Low household wealth has frequently been associated with poor sanitation and unsafe drinking water, as reported in the literature, and has been associated with stunting^(27)^.

According to national data for 2023, the percentage of people living below the poverty line in eastern Indonesia is generally higher than the national average (9·36 %)^(8)^. As these data showed that four of the five provinces with the highest poverty rates are located in eastern Indonesia, targeted economic development initiatives are essential. Furthermore, enhancing ownership of the national health insurance system is crucial to ensure that economically disadvantaged communities have access to high-quality maternal and child healthcare services. However, national insurance coverage in eastern Indonesia remains low^(24)^. There were four provinces located in this region, which had the lowest coverage of national health insurance. This indicates a strong need for targeted efforts to increase the enrolment and use of national insurance among communities in eastern Indonesia.

### Strengthening basic and advanced maternal and child healthcare services

As reported in previous studies, children born weighing less than 2500 g or before 37 weeks of gestation are more likely to experience stunting^(6,28)^. Therefore, low birth weight and preterm delivery should be reduced to ensure long-term health and development. Ensuring adequate maternal nutrition, regular antenatal care and managing other risk factors for low-birth-weight babies and preterm deliveries are essential^(29,30)^. The provision of quality healthcare services to vulnerable infants is vital. This should be a priority, particularly in eastern Indonesia, where disparities in healthcare services, facilities, medical professionals and essential supplies are frequently reported^(31)^. Prioritising essential medical interventions is critical to supporting the growth of these vulnerable infants, such as promoting optimal breast-feeding practices, fortifying breast milk with essential nutrients and implementing appropriate complementary feeding practices after exclusive breast-feeding^(32,33)^.

Our analysis aligns with previous studies, revealing a higher likelihood of stunting in infants from households with three or more children aged < 5 years^(26,34)^. Having several young children in a household often indicates a short interval between births, which can negatively affect a child’s health. This increases the demand for healthcare and the risk of infectious diseases, thereby compromising children’s nutritional status and growth potential. Improving the availability and access to family planning services is important to help families adequately space pregnancies and allocate sufficient time and resources to each child during their critical early years.

### Promoting effective health education interventions

As shown in another study, we found that children born to mothers with low education levels were more likely to experience stunted growth^(35)^. Limited knowledge of nutrition and health practices during pregnancy and early childhood, in addition to limited access to healthcare resources, may result in insufficient prenatal and postnatal care. Our result is consistent with previous literature that infants born to young mothers have an increased likelihood of stunting^(36,37)^. Pregnancy during adolescence leads to higher nutritional needs in adolescent girls who have not completed their growth and development. Therefore, pregnancy during adolescence leads to a struggle for nutrition between the mother and the fetus. This will create nutritional competition between the mother and fetus, often leading to conditions such as low birth weight and potential future health issues^(37)^. We also confirmed higher odds of stunting in male than female children, as reported in previous studies^(38)^. Male children are more susceptible to infections and undernutrition owing to sex-based biological differences.

This evidence highlights the need for effective health promotion strategies to raise awareness among parents, caregivers and the general community. In eastern Indonesia, limited access to quality education, a shortage of qualified teachers, inadequate resources, geographic isolation and economic constraints contribute to lower literacy rates and reduced opportunities^(39)^. Therefore, educating communities on proper nutrition, hygiene and healthcare practices can empower parents and caregivers to make informed decisions that support their children’s growth and development.

Our study also confirmed an increased likelihood of stunting in older children, a trend observed in other studies^(40)^. This might stem from poor nutrition and increased exposure to infections and illnesses in older children, which impair nutrient absorption and use. Therefore, increasing family awareness of the early detection of infant growth delays will allow timely intervention. Family awareness of growth milestones and signs of stunting encourages effective collaboration with healthcare providers to address issues promptly. Community-based programmes that disseminate information on the long-term impact of stunting are crucial for reducing stunting and improving child health outcomes in eastern Indonesia.

### Strengths and limitations

This study used nationally representative data from the Ministry of Health, including a large sample size suitable for examining the association between various factors and stunting in children aged < 2 years. A multilevel modelling approach was employed to investigate the significance of provinces and districts in reducing stunting. However, the use of cross-sectional data was a major limitation of this study, which precluded causal inferences. Furthermore, our analysis was constrained by the availability of variables in the dataset, excluding factors associated with stunting, such as nutritional diversity, diseases and infections, food security status and maternal stature. Additionally, the data relied on the mothers’ memories, which could have affected the accuracy.

### Conclusions

In summary, our study demonstrated a high prevalence of stunting in eastern Indonesia. Various factors, including community and child characteristics, contribute to stunting. This highlights the need for comprehensive initiatives to address immediate nutritional deficiencies and broader socio-economic challenges in eastern Indonesia. These initiatives could include strategies to alleviate poverty, improve the healthcare system and expand family planning services to help mothers manage their pregnancy intervals. Consistent and continuous health promotion programmes are crucial for raising community awareness of effective stunting prevention strategies and for monitoring children’s growth, especially among less-educated women. Policymakers and programme managers should prioritise localised interventions over broad province-wide strategies to address the unique conditions of each district. This will help optimise resource allocation and have a greater impact on reducing stunting rates in eastern Indonesia.

## Supporting information

Titaley et al. supplementary material Titaley et al. supplementary material

## Supplementary material

For supplementary material/s referred to in this article, please visit https://doi.org/10.1017/S0007114525105771
