# Supplementary material for: Stunting in children aged under 2 years living in the eastern part of Indonesia: analysis of the 2010–2018 Indonesia Basic Health Research
Source: Br J Nutr. 2025 Nov 26;135(2):221–31. doi: 10.1017/S0007114525105771 (PMC12885871; doi:10.1017/S0007114525105771)
Supplement: Titaley et al. supplementary material [file S0007114525105771sup001.docx]

**Supplementary Table 1**. The sample size, census block and number of households, The Indonesia Basic Health Research 2010-2018.

|  | **2010** | **2013** | **2018** |
| --- | --- | --- | --- |
| Sample size | 70.000 | 300.000 | 300.000 |
| Representation | Province | District | District |
| Number of Province | 33 | 33 | 34 |
| Number of District/City | 441 | 497 | 514 |
| Primary Sampling Unit (PSU) | Census Block | Census Block | Census Block |
| Number of PSU | 2.800 | 12.000 | 30.000 |
| Number of Household in each PSU | 25 | 25 | 10 |

**Supplementary Figure 1.** Proportion on stunting in children under two by district in West Nusa Tenggara Region, Indonesia, the 2013 and 2018 Indonesia Basic Health Research

**Supplementary Figure 2.** Proportion on stunting in children under two by district in East Nusa Tenggara Region, Indonesia, the 2013 and 2018 Indonesia Basic Health Research

**Supplementary Figure 3.** Proportion on stunting in children under two by district in Sulawesi Region, Indonesia, the 2013 and 2018 Indonesia Basic Health Research

**Supplementary Figure 4.** Proportion on stunting in children under two by district in Maluku Region, Indonesia, the 2013 and 2018 Indonesia Basic Health Research

**Supplementary Figure 5.** Proportion on stunting in children under two by district in Papua Region, Indonesia, the 2013 and 2018 Indonesia Basic Health Researc
